# Steady Beat Sound Facilitates both Coordinated Group Walking and Inter-Subject Neural Synchrony

**DOI:** 10.3389/fnhum.2017.00147

**Published:** 2017-03-27

**Authors:** Shigeyuki Ikeda, Takayuki Nozawa, Ryoichi Yokoyama, Atsuko Miyazaki, Yukako Sasaki, Kohei Sakaki, Ryuta Kawashima

**Affiliations:** ^1^Department of Ubiquitous Sensing, Institute of Development, Aging and Cancer, Tohoku UniversitySendai, Japan; ^2^Department of Functional Brain Imaging, Institute of Development, Aging and Cancer, Tohoku UniversitySendai, Japan; ^3^Japan Society for the Promotion of ScienceTokyo, Japan; ^4^Division of Developmental Cognitive Neuroscience, Institute of Development, Aging and Cancer, Tohoku UniversitySendai, Japan

**Keywords:** inter-subject neural synchrony (INS), walking, steady beat sound, wavelet transform coherence, functional near-infrared spectroscopy (fNIRS), frontopolar cortex

## Abstract

Group walking is a collective social interaction task as pedestrians are required to determine their own pace of walking on the basis of surrounding others’ states. The steady beat sound is known to be a controllable factor that contributes to relative success/failure of coordinated group walking since the beat improves pedestrian flow in congested situation. According to some reports, inter-personal interaction synchronizes inter-personal brain activity in the prefrontal region, which supports social cognitive processes required for successful inter-individual coordination, such as predicting each other’s state; success/failure of a coordinated task is associated with increase/decrease in inter-subject neural synchrony (INS). Combining these previous findings, we hypothesized that INS during group walking in congested situations would also differ depending on the existence of the steady beat, corresponding to the modulated coordination-related cognitive processes. Subjects’ frontopolar activities were measured using ultra-small near infrared spectroscopy, which can simultaneously measure the brain activities of multiple subjects without constraints on their motions. To exclude the possibility that increased INS may be spuriously induced by the shared stimuli (i.e., steady beat) or by the resultant behavioral synchronization, as control we used stepping on a same spot, which is similar in movement to walking but does not require the subjects to consider others’ states, either with or without the steady beat. In a two by two repeated measures factorial experimental design, the subjects were instructed to walk or keep stepping on a same spot with or without a steady beat sound of 70 beats per minute. As previously reported, the walking flow during group walking with the beat significantly increased compared with that without the beat. Synchronization of stepping between the subjects was also significantly increased by the steady beat sound. For INS, we observed a significant interaction effect between walking/stepping and sound/no-sound, supporting our hypothesis. INS while walking with the beat was higher than that without the beat, whereas the beat induced no significant differences in INS during stepping. Furthermore, the effect of the beat on INS while walking was spatially extended beyond the adjacent pedestrians, reflecting the diffuse nature of the collective coordination in group walking. The increase of INS for walking suggested that the steady beat sound led to more harmonized inter-personal cognitive processes, which resulted in the more coordinated group motion.

## Introduction

In a congested situation, a pedestrian needs to determine one’s own pace by interacting with and predicting other pedestrians’ states to avoid collisions. This issue has practical significance in evacuation during emergencies or in designing large social events (Helbing and Johansson, 2009). Auditory rhythm, such as metronome sound or music, is known to have a major influence on human walking (Styns et al., 2007). A previous study reported that pedestrian flow decreased during group walking in a congested situation, whereas the flow improved by playing a repeated sound from a metronome (70 beats per minute; BPM) to pedestrians (Yanagisawa et al., 2012). The fact indicates that the steady beat is a controllable factor that contributes success/failure of coordinated group walking, and suggests a possibility that the steady beat sound affects the pedestrians’ coordination-related cognitive processes for considering the state of other pedestrians in a congested situation. However, no studies have investigated the effect of a steady beat on such cognitive processes in pedestrians.

Inter-personal interactions synchronize inter-personal brain activity in the prefrontal region, which has a role in social interaction (Scholkmann et al., 2013). NIRS-based “hyperscanning” techniques have gained interest in inter-subject neural synchrony (INS) by measuring simultaneously multiple brains that socially interact with each other (Konvalinka and Roepstorff, 2012; Schilbach et al., 2013). A pioneering study using hyperscanning reported that INS between two subjects’ functional near-infrared spectroscopic (fNIRS) signals from the prefrontal cortex increased during a cooperative task in which the subjects simultaneously pressed their buttons (Funane et al., 2011); in contrast, INS did not increase during a competitive task in which the subjects pressed their buttons with the goal of responding faster than their partner (Cui et al., 2012). Furthermore, INS reportedly increased when two subjects more successfully interacted in the button-press task (Funane et al., 2011; Cui et al., 2012). Similarly, degree of relative successfulness of cooperative button-pressing by two subjects was reported to be associated with an increase/decrease in INS (Cheng et al., 2015). From these findings, brain-to-brain coupling is assumed to be caused by successful modeling, which is a learning process by observing behavior of the others, and prediction of the mental or physical states of peers during social interaction tasks. The button-press task shed light on the understanding of INS in the prefrontal region but the task setting is close to a laboratory experiment. Extending the previous findings into more real-life oriented social interaction, several studies showed connection between verbal communication and INS (Jiang et al., 2012, 2015; Nozawa et al., 2016). On the other hand, a previous study showed relationship between physical coordination (i.e., non-verbal interaction) and INS (Holper et al., 2012). The task used in the previous study was a dyadic coordination in which a subject imitated finger-tapping movements performed by the other, and thus the target to be coordinated was specific and focal. However, relationship between collective physical coordination, which is frequent in real-life and requires a more diffuse coordination with the surrounding, and INS remains unclear.

Group walking focused in the present study is a collective social interaction task as pedestrians are required to determine their own pace of walking on the basis of others’ states such as walking pace and physical distance between self and others. In addition, group walking is coordination with the surrounding crowd, where recognition of more than direct neighbors makes difference (Helbing and Molnar, 1995; Suma et al., 2012). Firstly, we confirm the previous result (Yanagisawa et al., 2012) that a steady beat sound (70 BPM) improved walking flow. Secondly and more essentially, we hypothesize that in congested group walking, a steady beat makes INS of the pedestrians’ prefrontal activities higher than that without the steady beat, reflecting the effect of steady beat in driving pedestrians into more successful modeling and prediction of each other’s state. Furthermore, we expect that the effect of the steady beat on the INS could be observed over a spatially extended range, reflecting the diffuse nature of the coordination where coordination with more than direct neighbors is expected to be an important factor for a successful group walking.

Frontopolar region, a part of the prefrontal cortex, was focused by the present study because the frontopolar region is known to be associated with social interaction (McCabe et al., 2001; Sänger et al., 2011). The frontopolar region showed higher activity during cooperative button-pressing compared to other prefrontal parts (Funane et al., 2011). Furthermore, significant INS related to the button-press task was observed in the frontopolar region; INS derived from the frontopolar region tended to increase with increasing task performance (Cheng et al., 2015).

**FIGURE 1:**
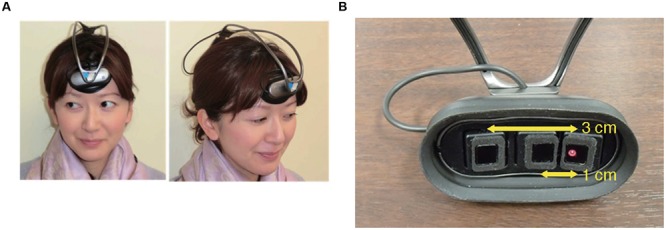
**Ultra-small near infrared spectroscopy (NIRS). (A)** Installation of an ultra-small NIRS. **(B)** The distance between the emitter and two detectors (1 and 3 cm, respectively).

INS may be susceptible to spurious relationships between the frontopolar activities and the external stimulus (i.e., the steady beat) or the resultant behavioral synchronization. To control such spurious relationships, we added stepping on a same spot, which is similar in movement to walking but does not require the subjects to consider others’ states, either with or without the steady beat. Therefore, our experiment consisted of four conditions: sound/no-sound walking and sound/no-sound stepping. We expected stepping did not show a significant difference of INS between presence and absence of the steady beat sound. To measure frontopolar activity, we used ultra-small NIRS (**Figure 1**; Nozawa et al., 2016) which has many advantages: multi-brain measurement, wireless, no limits for natural movement, and measurement of skin blood flow. To estimate INS, we used wavelet transform coherence (WTC) (Torrence and Compo, 1998; Grinsted et al., 2004). The obtained INS was statistically compared across two factors comprised of the four conditions (1st factor: walking/stepping, 2nd factor: sound/no-sound), using two-way repeated-measures analysis of variance (ANOVA). Expectation supporting our hypothesis was that INS during sound walking would become significantly higher than during no-sound walking, reflecting an interaction effect between the steady beat and group walking.

## Materials and Methods

### Subjects

The study included 97 healthy, right-handed university students (65 males and 32 females; age, 18-26 years; mean ± SD, 21.0 ± 1.6 years). The subjects had normal or corrected-to-normal vision and reported no history of neurological or psychiatric conditions. The subjects were recruited individually, and thus were mostly unacquainted with each other. Our study was approved by the Ethics Committee of Tohoku University Graduate School of Medicine and was conducted according to the Declaration of Helsinki. Written informed consent was obtained from all subjects.

### NIRS Instrumentation

To measure the subjects’ frontopolar activities, we used fNIRS, which exploits the relationship between neuronal metabolic activity and the oxygenation and concentration of hemoglobin in blood vessels (i.e., neurovascular coupling) (Ferrari and Quaresima, 2012). We used a wireless, portable, and ultra-small NIRS system (**Figure 1A**; Nozawa et al., 2016), which measures changes in the total hemoglobin (tHb) concentration with a light source of single wavelength at 810 nm. The sampling frequency of the device is 10 Hz. In this study, tHb concentration changes were used as brain activity in the analyses because of being far less sensitive to vein contamination and providing better spatial specificity (Dommer et al., 2012). The system does not require the subject’s body or head movement to be restrained and can simultaneously measure up to 10 subjects’ cerebral activities during a group action. The optical system comprises one light emitter and two light detectors; the emitter is 1 and 3 cm distant from the two detectors (**Figure 1B**); this is to enable our NIRS to create cerebrally unique responses by regressing out the nearby detector’s signals (1 cm) from that of the far detector (3 cm) (Saager et al., 2011). For recording, three optodes were placed over the subjects’ foreheads according to our previous implementation (Nozawa et al., 2016); these optodes approximately covered the center between FP1 and FP2 in the international 10–20 system, which correspond to the medial part of the frontal pole (Okamoto et al., 2004; Tsuzuki et al., 2007). Each subject’s hair under the optodes was carefully pushed off their foreheads. The optodes had a rubber cover to block out light.

### Experimental Conditions

**FIGURE 2:**
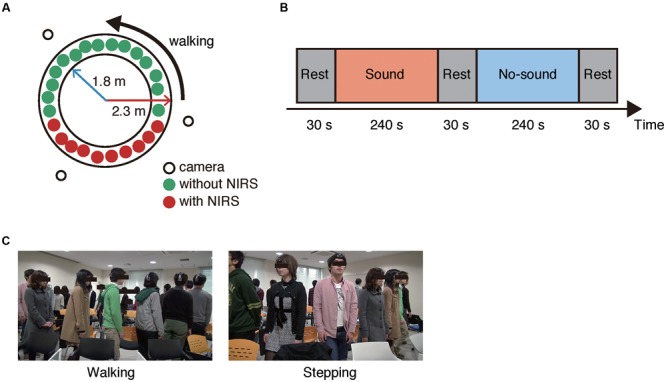
**Experimental setup. (A)** Experimental environment. Three video cameras were arranged around the circle. In each group, 10 randomly selected subjects (six males and four females) wore an ultra-small near infrared spectroscope and were sequentially positioned. All subjects walked in a counter-clockwise direction. **(B)** Block flow in a session. **(C)** Snapshots of the walking and stepping conditions.

Total of 97 subjects who were randomly assigned to four groups of 24 or 25 subjects (seven to nine females), participated in the experiment. Each group performed group walking with and without a steady beat sound (70 BPM) from a metronome. In the group walking conditions with or without the steady beat, the subjects were instructed to walk counter-clockwise on a circle with inner and outer radii of 1.8 and 2.3 m (**Figure 2A**) and to walk naturally keeping a smooth flow in mind (**Figure 2C**). In addition, while the steady beat sound was played, the subjects were instructed to perform walking along the beat. This procedure was the same as used in a previous study (Yanagisawa et al., 2012). The previous study defined degree of congestion in group walking as ρ = N/L (ρ: density, *N*: the number of pedestrians, *L*: length of circuit whose inner and outer radii were 1.8 and 2.3 m; Yanagisawa et al., 2012). According to their findings, the steady beat increased walking flow at ρ > 1.3; the effect was most significant at 1.8 ≤ ρ ≤ 2.0 (23 ≤ N ≤ 26), which gives the basis for our setting of “congested situation” in group walking.

As a control condition for group walking, the subjects performed group stepping, which was similar in movement to group walking but, unlike group walking, did not require consideration of others’ states. In stepping with and without the steady beat, the subjects were instructed to perform stepping along the steady beat or with their respective paces, respectively. Without focusing on the other subjects, each subject kept stepping on the same spot facing out in a radial direction from the circle (**Figure 2C**).

In our experiment, each subject group performed four sessions; in two of the four sessions, the groups performed either the group walking or the group stepping. Each session consisted of two task blocks (240 s) and three rest blocks (30 s; **Figure 2B**). The steady beat sound was played to the groups during one of the two task blocks. In sum, there were the four experimental conditions [i.e., sound walking (SW), no-sound walking (NW), sound stepping (SS), and no-sound stepping (NS)]. In each rest block, the subjects were instructed to stay as motionless as possible. The session order was counterbalanced across the groups, and the block order was counterbalanced across the sessions (Supplementary Table S1).

For the NIRS recording, 10 subjects (six males and four females) were randomly selected for each group; they wore an ultra-small NIRS and were sequentially positioned. To monitor the subjects’ behavior and evaluate the walking flow, three video cameras were installed outside the circle (**Figure 2A**).

### Evaluation of Walking Flow

We tested whether the steady beat sound actually improved walking flow, as has been shown in the previous study (Yanagisawa et al., 2012). Walking flow was defined as the number of people who passed a landmark in a unit time and was calculated as follows:

(1) $$\begin{array}{c} Q=\frac{n}{240}, \end{array}$$

where *Q, n*, and 240 denote the walking flow (person/second), the number of people who passed a landmark, and the constant time (in seconds) for each task block, respectively. Values of the walking flow calculated from three cameras in each session were averaged separately for SW and NW. To test whether the Q of SW significantly increased compared with that of NW, we applied a linear mixed-effects model with the fixed effects for the beat sound (sound/no-sound) and the sessions (1/2) and a random effect for the groups. The model was fitted using the restricted maximum-likelihood method and the denominator degrees of freedom was calculated using the Kenward-Roger’s method. The lme4 and lmerTest packages for R statistical software were used.

### NIRS Data Analysis

To remove slow drifts, the fNIRS signals were high-pass filtered using a set of discrete cosine basis functions with a cutoff period of 128 s (i.e., 7.8 × 10^-3^ Hz). To remove motion artifacts caused by walking and stepping, a wavelet-based motion artifact removal method (Molavi and Dumont, 2012) implemented in HOMER2^1^ was applied to the fNIRS signals.

To evaluate INS of all pairs of the subjects wearing ultra-small NIRS, we used WTC, a method of measuring the cross-correlation between two time series as a function of frequency or period as inverse of frequency and time (Torrence and Compo, 1998; Grinsted et al., 2004). Because WTC can show the local correlation between two time series for each frequency, it has been used in many fNIRS studies (Cui et al., 2012; Dommer et al., 2012; Holper et al., 2012; Jiang et al., 2012, 2015; Nozawa et al., 2016). We used a WTC Matlab package (Grinsted et al., 2004), which is provided on the authors’ website^2^.

We focused on the brain coherence value for each period between 10 and 85 s (i.e., 0.012–0.1 Hz) calculated by WTC because high- and low-frequency noise was removed and also in consideration of the cone of influence (COI). COI is defined at each period to avoid edge-effects that contaminate the estimates of wavelet coefficients and WTC at the boundaries between task and rest blocks (Torrence and Compo, 1998). An illustrative example of WTC between two fNIRS signals of a pair was shown in Supplementary Figure S1. The coherence value was time-averaged for each task block and averaged across two sessions in each condition (i.e., SW, NW, SS, and NS) for each pair of subjects. According to a previous study (Jiang et al., 2012), the coherence value between 0.01 and 0.1 Hz increased during face-to-face dialog, which could be considered as a type of coordinated social interaction task. Therefore, our periods of interest were supported by the previous findings. The upper bound of the period of interest (85 s or 0.012 Hz instead of 100 s or 0.01 Hz) was imposed by the duration 240 s of the task block and the COI (Supplementary Figure S1).

To investigate the interaction effects of two factors (1st factor: walking/stepping, 2nd factor: sound/no-sound) on brain coherence values, we used two-way repeated-measures ANOVA in which group was added as a covariate. There were 180 samples (number of combinations from 10 choose 2 = 45 pairs × 4 groups = 180) for each period and each condition; for each period, a *p*-value of the interaction effect between the two factors was acquired by computing ANOVA. To correct for multiple testing for all periods, we used the false discovery rate (FDR) approach (Benjamini and Hochberg, 1995) with a threshold of *q* ≤ 0.05.

The near detector (1 cm) of the ultra-small NIRS could measure skin blood flow, which allowed for the test of whether changes in INS were spuriously derived from motion artifacts during the task or activities in the autonomic nervous system. Using the same procedure as mentioned above, we investigated whether interaction effects of the two factors were observed in the coherence values calculated from skin blood signals.

## Results

### Behavioral Data

**FIGURE 3:**
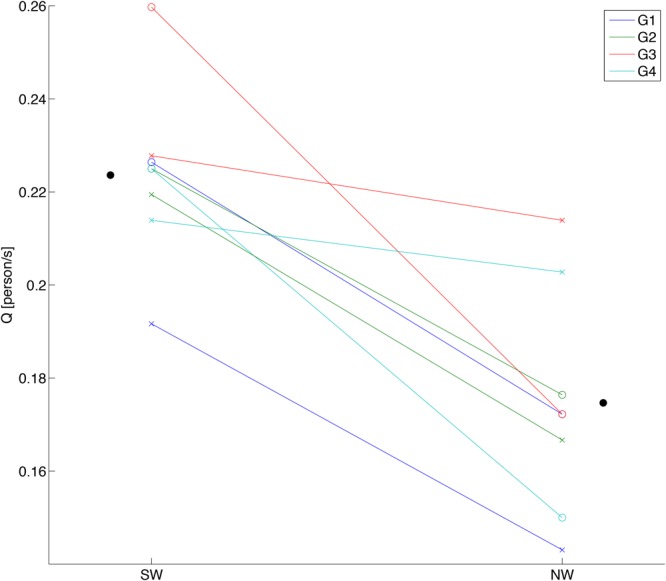
**Behavioral data of group walking.** Each data point shows a walking flow (*Q*) of each session in each subject group. SW and NW represent sound walking and no-sound walking, respectively. White circles show walking flows in first session, and cross marks show walking flows in second session. Black circles show the ground mean values calculated by averaging the walking flows over all groups and sessions for each condition.

For group walking flow *Q* for the SW was consistently higher than for the NW over all sessions of all groups (**Figure 3**). The statistical results assessed by estimating the mixed-effects model verified a significant main effect of the beat sound [*F*_(1,3)_ = 46.83, *p* = 6.39 *×* 10^-3^], while neither the interaction of the beat sound and the sessions nor the main effect of the sessions were significant [*F*_(1,3)_ = 5.89, *p* = 0.0936 and *F*_(1,3)_ = 0.10, *p* = 0.775, respectively]. This result indicated a positive effect of the steady beat sound on walking flow, which is in agreement with previous findings (Yanagisawa et al., 2012). For the stepping conditions, we tested whether the steady beat sound induced synchronization of stepping between the subjects (Supplementary Figure S2); the result showed that stepping with the steady beat was significantly synchronized between the subjects compared to stepping without the beat.

### Interaction Effect between the Steady Beat Sound and Group Walking

**FIGURE 4:**
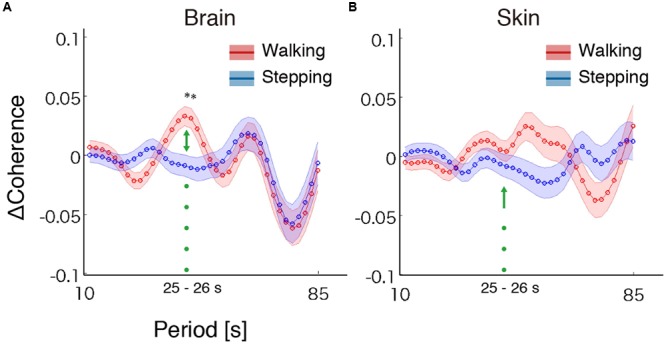
**Results of coherence analysis. (A)** Coherence differences when using cerebrally unique responses (brain) obtained by regressing out the skin blood flow. Each line and shading (red or blue) indicates the mean and the standard error of the mean of the coherence difference (Δ = sound–no-sound) during walking or stepping for all periods, respectively. Asterisks show the significant interaction effects by two-way repeated-measures analysis of variance (ANOVA; walking/stepping × sound/no-sound). To correct for multiple testing for all periods, false discovery rate correction (FDR, *q* = 0.05) was used. **(B)** Coherence differences when using skin blood flow data. No significant interaction effects were observed (FDR, *q* = 0.05).

To test our hypothesis, we examined whether the steady beat induced a difference in brain coherence only for the walking condition, by testing interaction effects between the two factors walking/stepping and sound/no-sound for each period. Multiple testing for all periods was corrected using a FDR (*q* = 0.05) correction. We observed significant interaction effects in the periods from approximately 25–26 s [period = 24.96 s, *F*_(1,176)_ = 11.01, *p* = 1.10 × 10^-3^, *q* = 0.02, $\eta_{G}^{2}$ = 0.015; period = 26.45 s, *F*_(1,176)_ = 10.71, *p* = 1.28 × 10^-3^, *q* = 0.02, $\eta_{G}^{2}$= 0.014] (for coherence values and simple main effects at the significant period 25 s, see **Tables 1, 2**). Generalized eta squared ($\eta_{G}^{2}$) represents the effect size in ANOVA. To clearly present the significant interaction effects, **Figure 4A** shows the coherence difference (sound–no-sound) for both group walking and group stepping; it also shows the significant interaction effects in the periods from approximately 25–26 s with asterisks. At the significant periods, the coherence differences of walking were positive values while the differences of stepping were nearly zero, supporting our hypothesis. In comparison, as shown in **Figure 4B**, the skin blood flow data exhibited no significant interaction effects in all periods.

**Table 1:** Brain and skin signals’ coherence values at the period ∼25 s.

Target	Experimental conditions	Mean	SEM
Brain	Sound walking	0.323	6.100 × 10^-3^
	No-sound walking	0.290	5.959 × 10^-3^
	Sound stepping	0.310	6.470 × 10^-3^
	No-sound stepping	0.319	6.401 × 10^-3^
Skin	Sound walking	0.300	5.805 × 10^-3^
	No-sound walking	0.295	6.376 × 10^-3^
	Sound stepping	0.288	6.079 × 10^-3^
	No-sound stepping	0.295	6.589 × 10^-3^

*Brain: brain signals obtained by regressing out the skin blood flow signals; Skin: skin blood flow signals; SEM: standard error of the mean.*

**Table 2:** Simple main effects of walking/stepping and sound/no-sound on brain and skin signals’ coherence values.

Target	Simple main effect	*F*_(1,176)_	*p* (^∗^ < 0.05)	$\eta_{G}^{2}$
Brain	F1 at sound	1.67	0.20	5.26 × 10^-3^
	F1 at no-sound	11.17	1.01 × 10^-3^∗^^	0.03
	F2 at walking	16.51	7.29 × 10^-5^∗^^	0.04
	F2 at stepping	1.00	0.32	2.66 × 10^-3^
Skin	F1 at sound	2.22	0.14	5.97 × 10^-3^
	F1 at no-sound	1.11 × 10^-3^	0.97	3.38 × 10^-6^
	F2 at walking	0.42	0.52	1.09 × 10^-3^
	F2 at stepping	0.52	0.47	1.52 × 10^-3^

*Using two-way repeated-measures ANOVA, we assessed whether there were significant simple main effects of walking/stepping (F1) and sound/no-sound (F2) on the coherence values at the period ∼25 s. Generalized eta squared ($\eta_{G}^{2}$) represents the effect size in ANOVA. Brain: brain signals obtained by regressing out the skin blood flow signals; Skin: skin blood flow signals. Asterisks indicate the significant simple main effects.*

### Spatial Distance Dependency of the Steady Beat Effect on the INS during Group Walking

**FIGURE 5:**
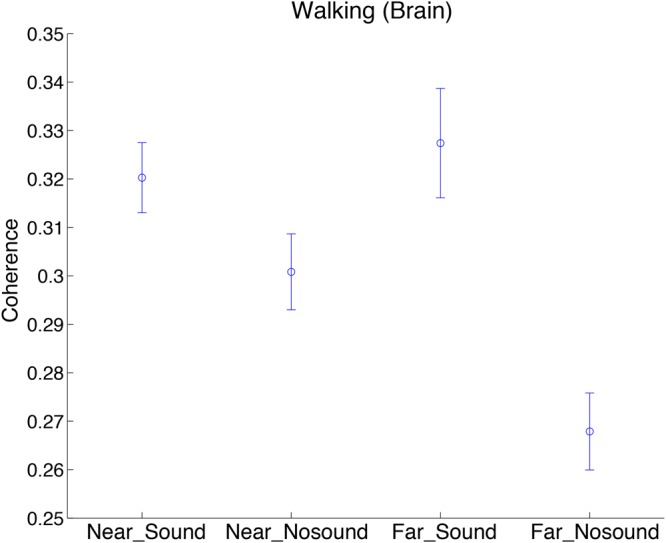
**Comparison of INS during walking in sound/no-sound conditions, divided by near/far pedestrian pairs.** Coherence values at the significant period *∼*25 s as a function of two factors (near/far and sound/no-sound) was plotted. “Brain” indicates brain signals obtained by regressing out the skin blood flow signals. Error bars show standard error of the mean.

To investigate the spatial nature of the observed steady beat effect on the INS in the walking conditions, we divided the 45 pairs in each subject group into “near” and “far” groups. Defining distance between two adjacent subjects as 1, near group consisted of pairs whose mutual distance was from 1 to 4, whereas far group consisted of pairs whose mutual distance was from 5 to 9. To investigate effects of the near/far factor and sound/no-sound factor on INS, we performed two-way repeated-measures ANOVA in which group was added as a covariate. INS during walking at the significant period ∼25 s as a function of two factors was shown in **Figure 5** (Supplementary Figure S3 for INS of stepping and skin blood flow signals). We observed a significant interaction effect of the two factors in walking [*F*_(1,172)_ = 5.83, *p* = 0.02; for simple main effects see **Table 3**]. In addition, we observed a significant main effect of the sound/no-sound factor [*F*_(1,172)_ = 22.66, *p* = 4.08 *×* 10^-6^] but not of the near/far factor [*F*_(1,172)_ = 1.92, *p* = 0.17]. No significant main effects or interactions were observed in the INS during stepping nor in the coherence of skin blood flow signals during walking or stepping.

**Table 3:** Simple main effects of near/far and sound/no-sound on brain signals’ coherence values in walking.

Simple main effect	*F*	*p*(^∗^ < 0.05)
Sound/no-sound at near	*F*_(1,116)_ = 3.94	0.0496^∗^
Sound/no-sound at far	*F*_(1,56)_ = 21.43	2.22 × 10^-5^∗^^
Near/far at no-sound	*F*_(1,172)_ = 7.08	8.53 × 10^-3^∗^^
Near/far at sound	*F*_(1,172)_ = 0.32	0.57

*Using two-way repeated-measures ANOVA, we assessed whether there were significant simple main effects of near/far and sound/no-sound on the coherence values at the period ∼25 s in walking. Asterisks indicate the significant simple main effects.*

## Discussion

In our study, ultra-small NIRS was used to measure frontopolar activities while the subjects performed group walking that is a collective social interaction, or group stepping. When a metronome sound of 70 BPM was played during group walking, the walking flow *Q* significantly increased compared with that during the no-sound condition. The steady beat sound also increased synchronization of stepping between the subjects. For INS, we observed significant interaction effects of the two factors (walking/stepping and sound/no-sound) at the period from approximately 25–26 s, indicating that the beat made INS while walking higher than that without the beat. This effect may reflect the individuals’ improved predictability of the collective physical coordination facilitated by the steady beat sound. Music that includes a steady beat or some beats is commonly used in difficult and complex group actions (e.g., dance). Therefore, our findings suggest a possible effect of music on frontopolar INS also during such group actions. In contrast, no significant interaction effects were observed in skin blood flow, supporting the cerebral origin of the observed interaction effects. Furthermore, we observed the effect of the beat on the INS over a spatially extended range, suggesting the diffuse nature of the coordination. Our study is the first to investigate relationship between collective physical coordination and INS, and to reveal the effect of the beat on INS.

The reason for introducing the stepping conditions was to verify whether the external auditory stimulus and resultant behavioral synchronization induce brain synchrony between the subjects, independently of coordination-related components. If the external stimulus induces brain synchrony between the subjects, INS during stepping with the beat is supposed to be higher than that during stepping without the beat. However, we did not observe such effect of the beat in stepping conditions. Therefore, the significant difference of INS between walking conditions (with and without the beat) is not attributable to the external stimulus.

The previous studies suggested that the coordination-related components of the task contributed to the increase of INS (Funane et al., 2011; Cui et al., 2012). This suggestion led to a question of whether INS for the walking conditions became higher than that for the stepping conditions. To investigate it, we confirmed simple main effects between the four conditions (SW, NW, SS, and NS) on the brain coherence at the significant period ∼25 s, using ANOVA (**Tables 1, 2**). Accordingly, INS for NW was lower than that for NS, and INSs between SW and SS did not show the significant difference. These results were inconsistent with the suggestion by the previous studies. A likely explanation for the phenomenon is that relative success/failure in coordination is another key factor in the increase/decrease of INS, in addition to the mere existence of demand for coordination in the task. The previous studies used coordinated social interaction tasks (e.g., simultaneous button press) in which subjects could easily cooperate with each other without support to help coordination (e.g., the steady beat). Unfortunately, such easy tasks may not dissociate the importance of the two factors. Our results indicate that even in a situation which involves high demand for coordination, relative failure in coordination (as in NW) could be accompanied by presumably high but uncoordinated recruitment of social processes, which can lead to even lower INS compared to the situations with no coordination demand (as in SS and NS). This interpretation can also reconcile the contrasting result obtained by Holper et al. (2012). They compared INS during a dyadic imitative coordination task between self-paced and stimulus-paced modes, and found higher INS during the self-paced mode compared to the stimulus-paced mode. This may have been because the self-paced mode imposed higher demand for coordination than the stimulus-paced mode, while the relative successfulness in coordination was not significantly different between the two modes (as indicated by “the mean inter-response duration” as their performance measure of coordination).

Furthermore, due to the diffuse nature of the collective coordination in group walking, we expect that the steady beat sound could affect the INS between pairs of pedestrians who are not spatially adjacent. The results of the analysis comparing INS between near/far pairs (**Figure 5, Table 3**, and Supplementary Figure S3) revealed that the presence or absence of the steady beat sound induced larger difference in INS between far pedestrian pairs rather than near pairs. This is consistent with our expectation, suggesting the possibility that the steady beat sound could facilitate virtual interaction between subjects who are distant from each other, which could improve collective pedestrian flow in crowded situations.

Frontopolar region is known to have a role in social interaction. We confirmed this by comparing the activation level of the measured frontopolar neural signals between the walking and stepping blocks. As a result, we observed significantly higher activation during walking than stepping (Supplementary Figure S4). The result indicated that the measured frontopolar neural signals indeed reflected functions involved in social interaction and coordination, of which demand should be higher during group walking. Therefore, we would speculate that the observed difference in INS induced by the presence/absence of steady beat during walking reflects temporally well-/ill-aligned recruitment of coordination-related cognitive processes, such as mentalizing and predicting each other’s states.

Our study, using ultra-small NIRS, provided a novel experimental paradigm that enabled multi-person studies, i.e., involving more than two subjects, of realistic social interactions in which the subjects had no limits for natural movement. Most previous studies on social interactions have focused on interactive tasks wherein the subjects had some constraints on their motions (NIRS: Cui et al., 2012; Jiang et al., 2012; Cheng et al., 2015; Duan et al., 2015; EEG: Lindenberger et al., 2009; Astolfi et al., 2010; Dumas et al., 2010; Yun et al., 2012) because the signal transfer was restricted by NIRS fibers or electric cords of the EEG. In an attempt to relieve such constraints, using a portable EEG, a previous study showed the possibility of measuring brain activities of multiple persons during interaction wherein the subjects had none of the above-mentioned limits (Gevins et al., 2012). However, EEG measurement is very susceptible to artifacts arising from muscle and eye movements; therefore, NIRS measurements may be considered more reliable for measuring brain activities under conditions having no movement constraints. Two previous studies used wireless NIRS, which overcomes the aforementioned limitations for EEG measurement during social interaction tasks (Dommer et al., 2012; Holper et al., 2012). However, these previous studies did not investigate unconstrained bodily social interactions between >2 subjects. A previous study investigated INS by verbal communication among three subjects using common NIRS with limits for subjects’ movement (Jiang et al., 2015). Our previous work showed the effectiveness of our ultra-small NIRS on studying INS by natural and unstructured verbal communication between four subjects (Nozawa et al., 2016). The present study, furthermore, extends the experimental paradigm of fNIRS hyperscanning to a next step that enables multi-person studies without limits for natural movement, i.e., group walking.

We observed the significant interaction effects in the period from approximately 25–26 s (i.e., 0.038–0.04 Hz). The low-frequency range (0.01–0.25 Hz) could be subdivided into four bands, as previously defined (Penttonen and Buzsáki, 2003; Buzsáki and Draguhn, 2004; Zuo et al., 2010): slow-5 (0.01–0.027 Hz), slow-4 (0.027–0.073 Hz), slow-3 (0.073–0.198 Hz), and slow-2 (0.198–0.25 Hz). Furthermore, gray matter-related hemodynamic oscillations have been reported to primarily occur in slow-4 and slow-5 (0.01–0.073 Hz) (Zuo et al., 2010). Because our significant results were observed between approximately 0.038–0.04 Hz within slow-4, the results were considered to have originated from the gray matter oscillations.

During the resting state, the low-frequency oscillation amplitudes in slow-5 are known to be dominant within medial prefrontal cortices (including part of the frontal pole) relative to that of slow-4 (Zuo et al., 2010). Unfortunately, the frequency dependency of cerebral functions remains unclear. Our results suggested that the oscillations within slow-4 in the frontal pole had an important role in a social interaction task (i.e., group walking). Future research is needed to investigate the functional significance of slow-4 and -5 in various social interactions.

Our NIRS system has limitations on the number of sensors and the coverage, i.e., the frontal pole only. Nevertheless, our findings of relationship between the coordinated group walking, steady beat sound, and INS obtained using the system are novel. Furthermore, the frontal regions, including the frontal pole, have been assumed to be important for social interactions [i.e., the frontal pole (McCabe et al., 2001; Cheng et al., 2015); the right superior frontal cortex (Cui et al., 2012); the left prefrontal region (Dommer et al., 2012), and left inferior frontal cortex (Jiang et al., 2012)]. Therefore, we suggest that our NIRS covering the frontal pole may contribute to better understanding of the neural mechanisms of social cognition. On the other hand, larger number of sensors and wider coverage may bring additional useful information because brain activities related to social interaction are widely distributed in the brain, such as in the temporal regions (Lindenberger et al., 2009) and right parietal region (Dumas et al., 2010).

## Conclusion

The present study using NIRS-based hyperscanning revealed that a metronome sound improved pedestrian flow and INS, and that the steady beat sound has an effect of extending range where pedestrians physically coordinate with other pedestrians. The obtained results promise to extend understanding of relationship between physical coordination and INS.

## Author Contributions

SI, TN, and RK designed the research. SI, TN, RY, AM, YS, and KS performed the experiments. SI analyzed the data. SI and TN wrote the manuscript. All authors reviewed the manuscript.

## Conflict of Interest Statement

The authors declare that the research was conducted in the absence of any commercial or financial relationships that could be construed as a potential conflict of interest.

## Footnotes

1 http://www.nmr.mgh.harvard.edu/PMI/resources/homer2/home.htm

2 http://noc.ac.uk/using-science/crosswavelet-wavelet-coherence
