# Supplementary material for: Steady Beat Sound Facilitates both Coordinated Group Walking and Inter-Subject Neural Synchrony
Source: Front Hum Neurosci. 2017 Mar 27;11:147. doi: 10.3389/fnhum.2017.00147 (PMC5366316; doi:10.3389/fnhum.2017.00147)
Supplement: Supplementary file 1 [file Table_1.DOCX]

**Table S1. Session order and block order.**

| Group | Session | 1^st^ block | 2^nd^ block |  |
| --- | --- | --- | --- | --- |
| 1 | 1. Walking  2. Stepping  3. Walking  4. Stepping | Nosound  Sound  Sound  Nosound | Sound  Nosound  Nosound  Sound |  |
| 2 | 1. Stepping  2. Walking  3. Walking  4. Stepping | Nosound  Sound  Nosound  Sound | Sound  Nosound  Sound  Nosound |  |
| 3 | 1. Walking  2. Stepping  3. Walking  4. Stepping | Nosound  Sound  Sound  Nosound | Sound  Nosound  Nosound  Sound |  |
| 4 | 1. Stepping  2. Walking  3. Walking  4. Stepping | Nosound  Nosound  Sound  Sound | Sound  Sound  Nosound  Nosound | |
