# Supplementary material for: Steady Beat Sound Facilitates both Coordinated Group Walking and Inter-Subject Neural Synchrony
Source: Front Hum Neurosci. 2017 Mar 27;11:147. doi: 10.3389/fnhum.2017.00147 (PMC5366316; doi:10.3389/fnhum.2017.00147)
Supplement: Supplementary file 2 [file Image_1.PDF]

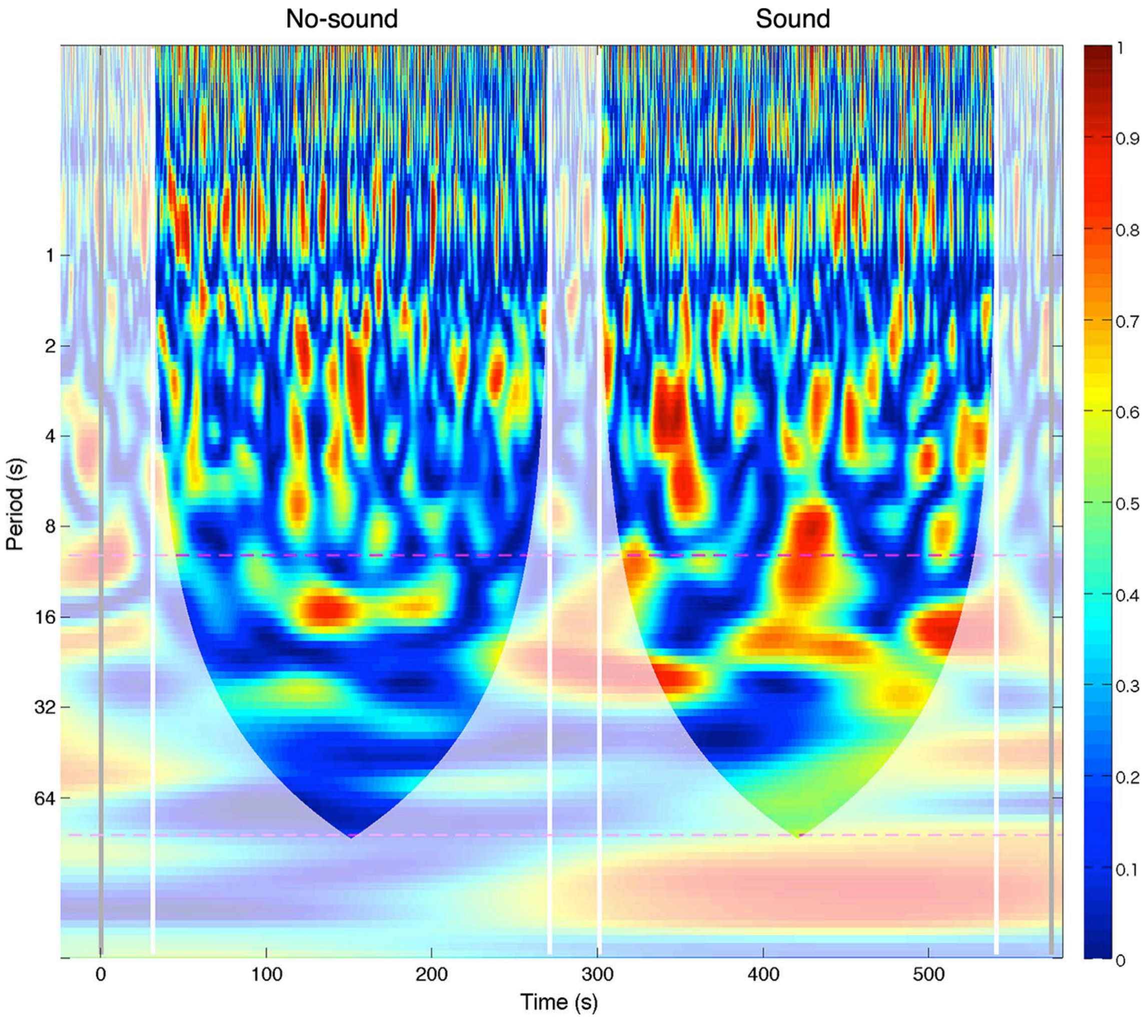

Figure S1: An example of wavelet transform coherence (WTC) during walking session.

WTC was calculated for two preprocessed frontopolar neural signals of each pair of subjects. Vertical grey lines indicate time points of session start and end (i.e., 0 s, 570 s). Vertical white lines indicate boundaries between task (240 s) and rest (30 s) blocks. Horizontal magenta dashed lines indicate boundaries of the focused periods (i.e., 10–85 s). The faded color areas bordered by spindle-like curves represent the cones of influence (see main text for detail).
