# Supplementary material for: Steady Beat Sound Facilitates both Coordinated Group Walking and Inter-Subject Neural Synchrony
Source: Front Hum Neurosci. 2017 Mar 27;11:147. doi: 10.3389/fnhum.2017.00147 (PMC5366316; doi:10.3389/fnhum.2017.00147)
Supplement: Supplementary file 3 [file Image_2.PDF]

Evaluating an effect of the steady beat sound on stepping

We tested whether the steady beat sound induced synchronization of stepping between the subjects. To quantitatively evaluate degree of stepping synchronization, audio data of stepping recorded by the video cameras was used. If stepping was synchronized with the steady beat, the audio signals by stepping should have only a component at a specific frequency (i.e. 70BPM ≈ 1.2 Hz). Conversely, since in stepping without the beat the subjects performed the task with their respective paces, the audio signals should have components over broader frequency range. If the assumption is correct, power components in stepping with the beat should show lower values than that of stepping without the beat in most frequencies except for the beat frequency of 1.2 Hz (and its harmonic frequencies). Note that comparison of the signal power only at the beat frequency of 1.2 Hz is not valid, because the sound of beat sound itself was also contributing in the steady beat condition. To confirm the assumption, the audio signals by stepping with and without the beat were rectified and then transformed to a frequency domain with a fast Fourier transform and the amplitude spectra were then acquired; a median of a distribution of the amplitude spectra within a frequency range from 0 to 5 Hz was calculated for each stepping condition. This range was determined with consideration for feasible number of steps in real stepping, i.e., 5 steps per second is an upper limit. The medians calculated from the three cameras and two sessions were averaged separately for two stepping conditions. We presented the medians of the subject groups for each stepping condition (Supplementary Figure 2). To investigate if the median of stepping with the beat was lower than that of stepping without the beat, a two-tailed paired t-test was performed ($t_3 = -4.15$, $p = 0.03$). From the result, we concluded that stepping with the steady beat was significantly synchronized between the subjects compared to stepping without the beat.

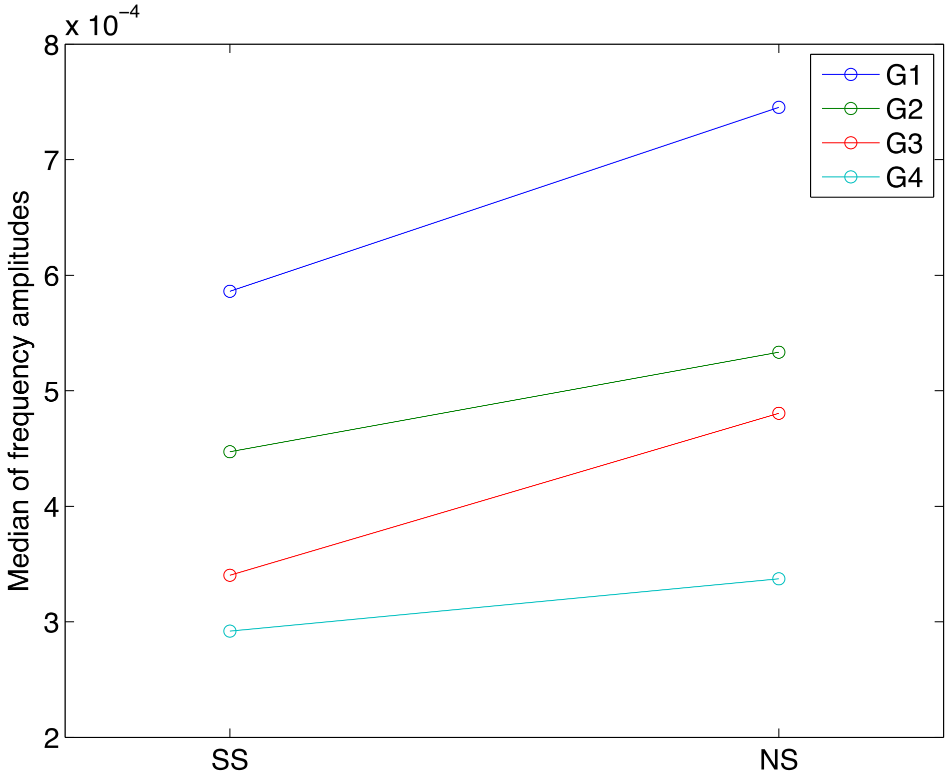

Figure S2: Effect of the steady beat sound on stepping

Each data point shows a median of a distribution of the amplitude spectra within a frequency range from 0 to 5 Hz. G1–4 show the subject groups and SS and NS show the stepping conditions, i.e., sound stepping and no-sound stepping.
