# Supplementary material for: Steady Beat Sound Facilitates both Coordinated Group Walking and Inter-Subject Neural Synchrony
Source: Front Hum Neurosci. 2017 Mar 27;11:147. doi: 10.3389/fnhum.2017.00147 (PMC5366316; doi:10.3389/fnhum.2017.00147)
Supplement: Supplementary file 4 [file Image_3.PDF]

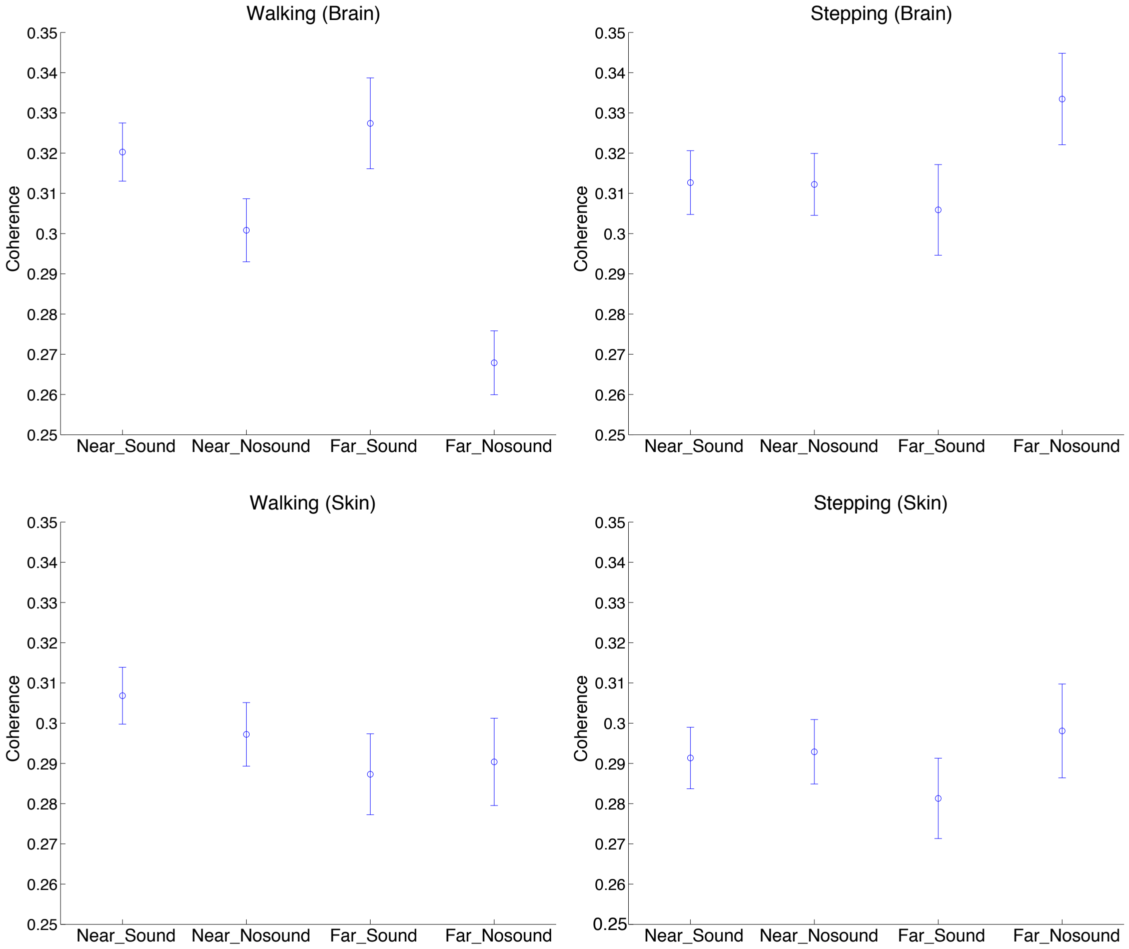

Figure S3: Coherence of walking/stepping on near/far and sound/no-sound. Coherence values at the significant period ~ 25 s as a function of two factors (near/far and sound/no-sound) were plotted. "Brain" indicates brain signals obtained by regressing out the skin blood flow signals. "Skin" indicates skin blood flow signals. Error bars show standard error of the mean. Two-way repeated-measures ANOVA in which group was added as a covariate was performed to investigate effects of the factors on the coherence values. In brain signals during walking, we observed a significant interaction effect of the two factors ($F_{(1,172)}$ = 5.83, p = 0.02) and a significant main effect of the sound/no-sound factor ($F_{(1,172)}$ = 22.66, p = 4.08 $\times 10^{-6}$) but not of the near/far factor ($F_{(1,172)}$ = 1.92, p = 0.17). No significant main effects or interactions were observed in the coherence of brain signals during stepping nor in the coherence of skin blood flow signals during walking or stepping.
