# Supplementary material for: Steady Beat Sound Facilitates both Coordinated Group Walking and Inter-Subject Neural Synchrony
Source: Front Hum Neurosci. 2017 Mar 27;11:147. doi: 10.3389/fnhum.2017.00147 (PMC5366316; doi:10.3389/fnhum.2017.00147)
Supplement: Supplementary file 5 [file Image_4.pdf]

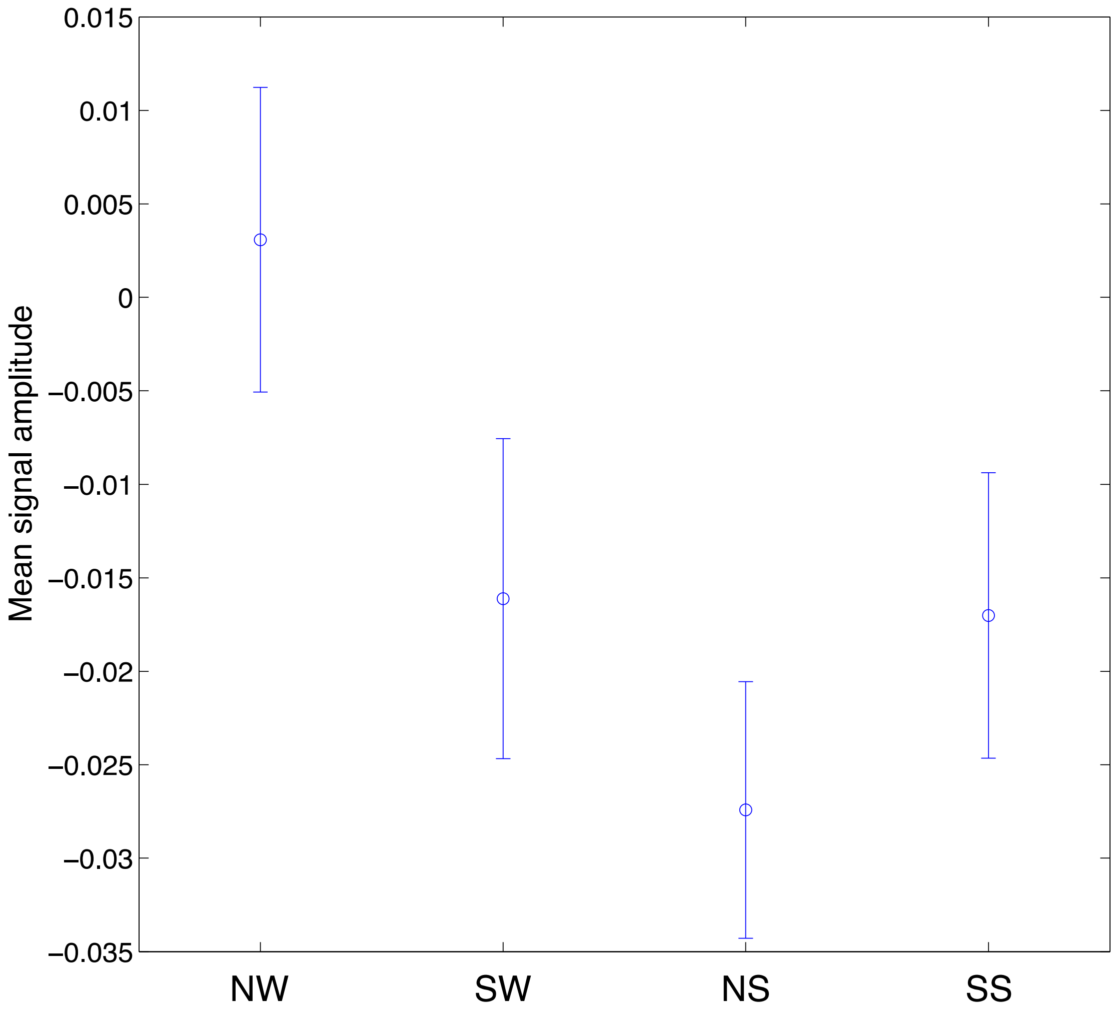

Figure S4: Mean signal amplitudes (Task – Rest) for four conditions.

In each session we calculated a mean signal amplitude from the neural signals during each task block and subtracted a mean signal amplitude calculated from rest blocks, from the mean signal amplitude of each task block. The mean values were averaged across sessions separately for the four conditions [i.e., no-sound walking (NW), sound walking (SW), no-sound stepping (NS), and sound stepping (SS)]. If measured neural signals reflect activations related to function in social interaction, the mean values of SW and NW are supposed to be higher than that of SS and NS. To confirm main effects on two factors (1st factor: walking/stepping, 2nd factor: sound/no-sound), we performed two-way repeated-measures ANOVA in which subject group was added as a covariate. We observed a significant main effect on 1st factor ($F_{(1,36)} = 4.82$, $p = 0.03$, $\eta_G{}^2 = 0.025$) but not on 2nd factor. In addition, we observed a significant interaction effect of the two factors ($F_{(1,36)} = 4.82$, $p = 0.03$, $\eta_G{}^2 = 0.022$). Generalized eta squared ($\eta_G{}^2$) represents

the effect size in ANOVA. Error bars show standard error of the mean.
